# Clinical outcomes and risk factors of hepatopulmonary syndrome in children

**DOI:** 10.1038/s41598-021-83785-x

**Published:** 2021-02-18

**Authors:** Kwang Yeon Kim, Tae Hyeong Kim, Jeong-Moo Lee, Nam-Joon Yi, Hyun-Young Kim, Jin Soo Moon, Jae Sung Ko

**Affiliations:** 1 Department of Pediatrics, Seoul National University College of Medicine, 101 Daehak-ro, Jongno-Gu, Seoul, 110-769 Korea; 2 Department of Surgery, Seoul National University College of Medicine, Seoul, Korea

**Keywords:** Gastroenterology, Risk factors

## Abstract

Hepatopulmonary syndrome (HPS) is defined as three distinct features: liver disease, hypoxemia, and intrapulmonary vasodilation. The purpose of this study was to investigate the clinical outcomes of pediatric HPS and to identify the risk factors for HPS in children with biliary atresia (BA). We performed a retrospective cohort study of all children who were diagnosed with HPS between 2000 and 2018 at Seoul National University Hospital. The clinical features and outcomes of the 10 patients diagnosed with HPS were reviewed. To clarify the risk factors of HPS in patients with BA, we reviewed 120 patients diagnosed with BA. Underlying liver disease was BA in 8 patients, portal vein agenesis in 1 patient, and portal vein thrombosis in 1 patient. A total of 7 patients underwent liver transplantation (LT). Currently, all seven patients, including 3 patients with severe HPS, survived after LT. The prevalence of HPS in children with BA was 7%. Polysplenia/interrupted inferior vena was the only risk factor for HPS in BA patients in multivariate analysis. The Pediatric End-Stage Liver Disease score was not associated with the development of HPS. Children with severe HPS undergoing LT had excellent outcomes. Screening for HPS in children with BA is required regardless of the severity of liver diseases.

## Introduction

Hepatopulmonary syndrome (HPS) is a complication that can result from liver cirrhosis or portal hypertension. This causes progressive symptoms such as dyspnea and cyanosis. The precise mechanism of HPS development is not fully understood. Increased bacterial translocation and endotoxin in cirrhosis leads to overproduction of vasodilatory substances such as nitric oxide (NO)^1^. Increased NO and pulmonary angiogenesis result in dilated pulmonary capillaries and ventilation–perfusion mismatch^2,3^.

HPS has three distinct features: liver disease, hypoxemia, and intrapulmonary vasodilation^4^. Alveolar-arterial oxygen gradient (PA-aO_2_) measurements are accurate measurements of hypoxemia. However, arterial blood gas analysis (ABGA) is impractical for pediatrics and is accompanied by complications such as arterial spasm and hematoma. For screening patients with HPS with cirrhosis, pulse oximetry has been validated as a screening tool. The threshold of a pulse oximeter ≤ 97% has been shown to detect PaO_2_ levels below 70 mm Hg with 100% sensitivity and 65% specificity^5,6^. Two methods can be used to identify intrapulmonary vasodilation: contrast enhanced echocardiography (CEE), most often using microbubbles as the contrast, and radioactive lung perfusion scan using macroaggregated albumin (MAA)^7^.

An effective medical therapy for HPS has yet to be established. Liver transplantation (LT) remains the only effective treatment for HPS. The occurrence of HPS is associated with increased morbidity and mortality^8,9^. In particular, HPS is a progressive disease and the outcome is poor if the degree is severe before LT. However, HPS is not considered a priority for LT in Korean children. Until recently, there are few studies on the risk factors and long-term outcome of HPS in children with BA.

The purpose of this study was to investigate the clinical outcomes of pediatric HPS and to identify the risk factors for HPS in children with BA in Korea.

## Materials and methods

We performed a retrospective cohort study of all children who were diagnosed with HPS between 2000 and 2018 at Seoul National University Hospital. HPS was diagnosed as having hypoxia with pulse oximetry less than 97% and intrapulmonary vasodilation among the patients with liver disease. Intrapulmonary vasodilation can be diagnosed by the CEE test when the microbubble is seen in the left ventricle within 4–6 heartbeats or when the shunt ratio of the brain exceeds 6% by the MAA test^10–12^. The shunt ratio evaluated by MAA was divided into three groups according to severity: mild (< 20%); moderate (20–40%); and severe (> 40%)^13–15^. The medical records of the 10 patients diagnosed with HPS were reviewed. These include gender, age, underlying liver disease, Child–Pugh–Turcotte (CPT) classification, Pediatric End-Stage Liver Disease (PELD) score, Model for End-Stage Liver Disease (MELD) score, pulse oximetry, chest X-ray, abdominal computerized tomography, and abdominal ultrasonography^16^.

To identify risk factors of HPS in patients with BA, we reviewed the medical records of 120 patients diagnosed with biliary atresia between 2000 and 2018 at Seoul National University Hospital. Univariate and multivariate analyses were used to investigate the risk factors, and a P value of < 0.05 was considered significant. The LT-free survival rate was analyzed through Kaplan–Meier survival analysis. Statistical analyses were performed with SPSS version 23.0 (IBM, New York, NY, USA). The study was performed in accordance with the Helsinki Declaration and approved by the ethical standards of the Institutional Review Board of Seoul National University Hospital (IRB No. 1808-136-967). Informed consent was waived by the Institutional Review Board of Seoul National University Hospital because the clinical data were obtained retrospectively.

## Results

### Baseline characteristics and outcomes of HPS patients

A total of 10 patients were diagnosed with HPS (Table 1). Five patients were male, and five patients were female. The median age at diagnosis of HPS was 11 years (range 1.5–22.3 years). Underlying liver disease was biliary atresia in 8 patients, portal vein agenesis in 1 patient, and portal vein thrombosis in 1 patient. The prevalence of HPS according to the underlying disease was 7% of patients with BA (8 out of 120), 20% of patients with portal vein agenesis (1 out of 5), and 5% of patients with portal vein thrombosis (1 out of 25). When classified as CPT classification, 4 were class A, 5 were class B, and 1 was class C. The median oxygen saturation by pulse oximetry was 87% (range 74–96%). Seven patients complained of dyspnea, and six patients had cyanosis. Three patients showed pulmonary vascular enlargement on chest X-ray. There were 6 positive cases in CEE and 7 positive cases in MAA. Three of them showed positive results in both tests. The median shunt ratio of the brain measured by MAA was 60%. In seven patients undergoing MAA, the severity of HPS was classified as one in the mild group, two in the moderate group and four in the severe group.

**Table 1:** Clinical details of patients with hepatopulmonary syndrome.

Case	Gender	Age at diagnosis of HPS (years)^B^	Liver disease	Polysplenia	IVC interruption	CPT Class	PELD/MELD	CEE	MAA (brain uptake %)^A^	Age at LT (years)^B^	Outcome	Follow-up duration (years)^B^
1	F	13.1	Biliary atresia			B	13	ND	89.0	13.1	LT	9.0
2	F	15.7	Biliary atresia			B	16	+	20.3		Waiting for LT	1.6
3	F	6.4	Biliary atresia			A	0	+	60.0	9.5	LT	8.3
4	F	1.5	Biliary atresia	+	+	A	0	+	ND		Died of UGI bleeding	4.3
5	M	1.6	Biliary atresia	+	+	A	0	ND	74.7	3.6	LT	8.7
6	F	13.6	Biliary atresia	+	+	C	23	ND	14.3	13.7	LT	5.7
7	M	22.3	Biliary atresia			B	8	+	32.8	22.6	LT	1.7
8	M	8.2	Biliary atresia			B	1.6	ND	63	10.4	LT	15.4
9	M	13.9	Portal vein agenesis		+	A	0	+	ND		Shunt surgery, stent	15.0
10	M	9.0	Portal vein thrombosis			B	5.6	+	ND	18.5	LT	12.3

*CEE* contrast echocardiography, *CPT* Child–Pugh-Turcotte, *HPS* hepatopulmonary syndrome, *IVC* inferior vena cava, *LT* liver transplantation, *MAA* macroaggregated albumin, *MELD* Model for End-Stage Liver Disease, *ND* not done, *PELD* Pediatric End-Stage Liver Disease, *UGI* upper gastrointestinal.

^A^Absolute number (percentage).

^B^Absolute number (years).

The median follow-up period from HPS diagnosis to the present was 8.5 years (range 1.6–15.4 years). Among 10 patients with HPS, 3 patients did not receive LT. One patient with portal vein agenesis underwent stenting insertion after splenorenal shunt surgery. One patient with BA waited for LT, and the other with BA died of uncontrolled upper gastrointestinal bleeding while waiting for LT. Only one of the total HPS patients died during the 10-year observation period, resulting in a 90% survival rate. The 10-year LT-free survival rate in HPS patients was 10%.

A total of 7 patients underwent LT. The median period from diagnosis of HPS to LT was 2 years (range 0.1–9.6 years). The median age of LT was 13.1 years (range 3.6–22.6 years). Among the LT recipients, 6 received living donor LT, and 1 received deceased donor LT. Currently, all seven patients are alive, and presenting symptoms improved after LT. Surgical complications, including vascular and biliary problems, were not noted after LT. Three patients with severe HPS underwent follow-up MAA after LT (median 7.7 months, range 5.6–8.2 months). They all had less than 6% uptake in the brain (median 2%, range 0–4.6%).

### Risk factors and outcomes of HPS in children with BA

A total of 112 patients were diagnosed BA without HPS (Table 2). The median oxygen saturation by pulse oximetry was 100% (range 97–100%). None of the patients complained of dyspnea, and none had cyanosis. The median follow-up period from birth to the present was 9.6 years (range 0.5–19.0 years). Among 30 patients who did not receive LT, 4 patients died. A total of 82 patients underwent LT. The median age of LT was 1 year (range 0.2–15.2 years). Among the LT recipients, 54 received living donor LT, and 28 received deceased donor LT. Currently, only two of the total LT patients with non-HPS BA died during the observation period, resulting in a 97.5% survival rate.

**Table 2:** Comparison of clinical manifestation and outcomes between BA with HPS and BA without HPS.

	BA without HPS (n = 112)	BA with HPS (n = 8)
**Clinical manifestation**		
Dyspnea	0	7
Cyanosis	0	6
SpO_2_, %, median (range)	100 (97–100)	87 (74–96)
Age at LT, years, median (range)	1.0 (0.2–15.2)	11.7 (3.6–22.6)
Post-LT survival	80/82 (97.5%)	6/6 (100%)
Non-LT survival	26/30 (86.7%)	1/2 (50%)

*LT* Liver transplantation.

Table 3 summarizes the risk factors for HPS in children with BA. PELD and MELD scores were not associated with the development of HPS. Varix and polysplenia/interrupted inferior vena cava (PS/IVC) were associated with an increased risk of HPS in the univariate analysis (*p* = 0.005 and *p* = 0.001, respectively). Among 8 BA patients with HPS, 3 (38%) had PS/IVC. In contrast, of 112 BA patients without HPS, 1 had PS/IVC. In the multivariate analysis, PS/IVC was a risk factor for HPS in children with BA (*p* = 0.001). Interrupted IVC was observed in a patient with portal vein agenesis.

**Table 3:** Univariate and multivariate analysis of risk factors for hepatopulmonary syndrome in children with BA.

	Univariate			Multivariate		
	OR	95% CI	*p value*	OR	95% CI	*p value*
Male gender	1.16	0.26–5.10	0.84			
Age	1.03	0.91–1.18	0.63			
PELD	0.84	0.71–1.00	0.050			
MELD	0.66	0.24–1.81	0.422	–	–	–
Varix	22.04	2.59–187.22	0.005	–	–	**–**
Congenital cardiac anomaly	0.23	0.40–1.33	0.101	**–**	**–**	**–**
Polysplenia/interrupted inferior vena cava	66.60	5.84–759.61	0.001	142.66	4.59–4433.76	0.005

*MELD* Model for end-stage liver disease, *OR* odds ratio, *PELD* pediatric end-stage liver disease.

**Figure 1:**
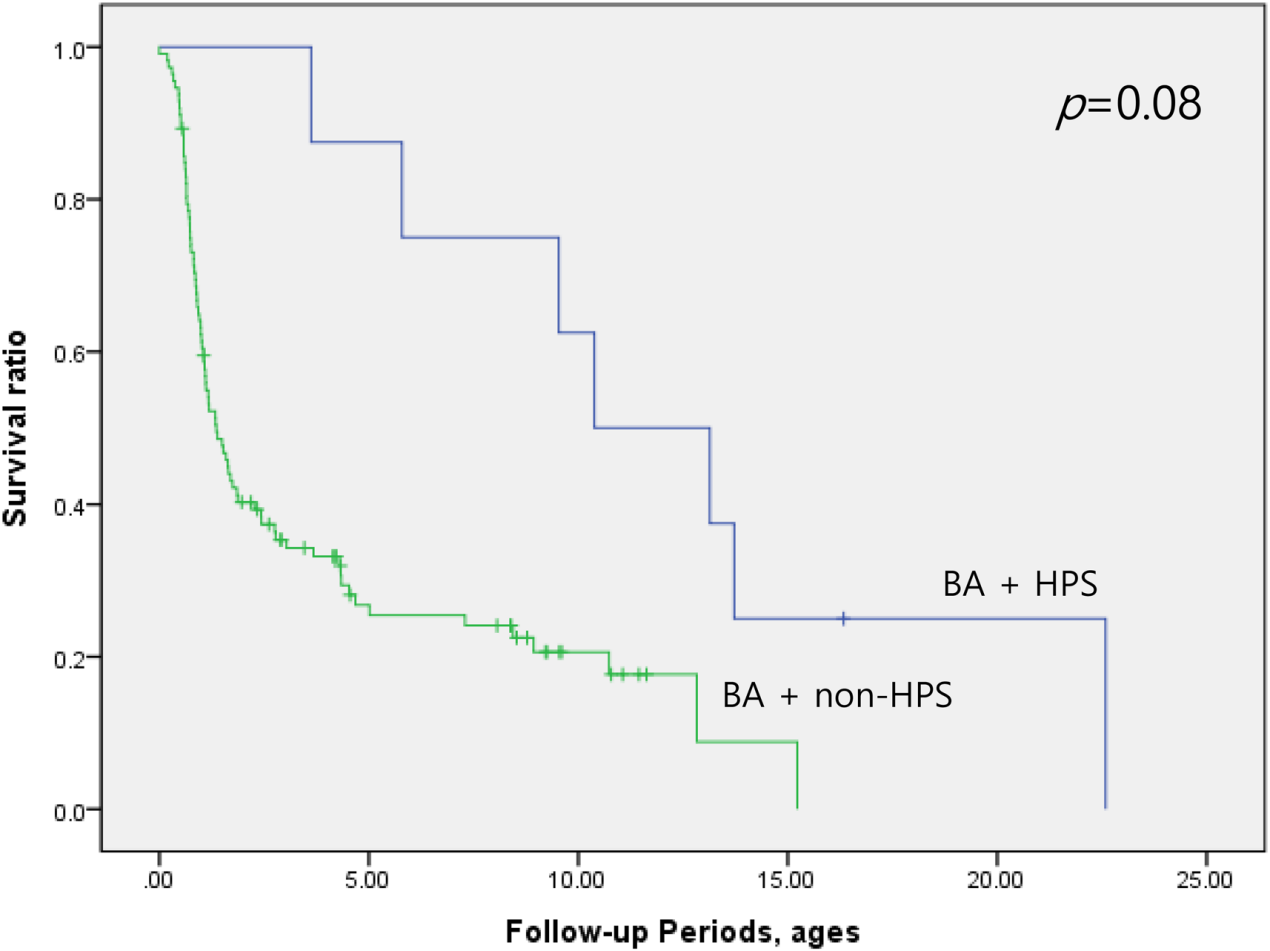
Comparison of 10-year LT-free survival rate between BA with HPS and BA without HPS. The Kaplan–Meier survival analysis showed that the 10-year LT-free survival rate did not differ significantly between BA patients with and without HPS (*p* = 0.08).

BA patients with HPS underwent LT at older age (median 11.7 years, range 3.6–22.6 years) than BA patients without HPS (median 1.0 year, range 0.2–15.2 years). Kaplan–Meier survival analysis showed that 10-year LT-free survival rate did not differ significantly between BA patients with and without HPS (p = 0.08) (Fig. 1).

## Discussion

In our study, the prevalence of HPS in children with BA, portal vein agenesis, and portal vein thrombosis were 7%, 20%, and 4%, respectively. The prevalence of HPS in adults with liver cirrhosis ranged from 4 to 29%^8,17^. In children, the prevalence of HPS was 3–20% in biliary atresia and 0.5% in portal vein thrombosis^16,18,19^. Previous studies have shown that the prevalence of HPS in children with PS/IVC or cirrhosis is between 6.1% and 42.5%^19,20^. The reason for this difference is that the types of liver disease and the method of diagnosing HPS were different in each study^16,21^.

To diagnose HPS, we followed the current well-known diagnostic criteria^11,22–24^. HPS was diagnosed as having hypoxia with pulse oximetry less than 97% and intrapulmonary vasodilation through CEE or MAA among the patients with liver disease. We did not perform ABGA to measure the PA-aO_2_. In children, ABGA is considered to be invasive, and pulse oximetry is an appropriate alternative to ABGA^16^. We measured pulse oximetry periodically and performed additional tests when pulse oximetry was lower than 97%. CEE or MAA was performed to diagnose intrapulmonary vasodilation. In children, MAA is known to have higher sensitivity for identifying intrapulmonary shunts than CEE^2,25^. MAA can be used to quantify and follow the degree of shunting^10,26,27^. The moderate-severe group accounted for 85% of our seven patients who underwent MAA, and all of them survived.

There is a debate over whether the severity of hepatic dysfunction is associated with HPS. Tumgor et al. reported that the PELD score and the CPT classification were associated with the development of HPS^28^. In our study, PELD and MELD scores were not risk factors for HPS. This finding is consistent with previous studies showing that there is no correlation between the presence or severity of HPS and the severity of liver disease based on the CPT classification, PELD or MELD score^29,30^.

HPS is known as a progressive disease when it occurs. The need for oxygen in a patient's daily life negatively affects the quality of life of children and adolescents, and HPS can be life threatening if not treated^9^. Previous studies have shown that if PaO_2_ is reduced below 50 mmHg, the prognosis is poor even after LT^30^. PaO_2_ is not included when calculating PELD and MELD scores. For this reason, the PELD and MELD scores do not increase correspondingly even if HPS develops and worsens. In our study, PELD and MELD scores were not high in patients with HPS. Our patients with HPS had a longer period from diagnosis to LT compared with those in the previous study^9^, and most of them had living donor LT. We found that BA patients with HPS underwent LT at older age than those without HPS, and 10-year LT-free survival rate did not differ significantly between BA patients with and without HPS. In previous studies, there was no difference in survival rate between patients who received LT in HPS patients and those who received LT in patients without HPS. However, if patients with HPS do not receive LT, their survival rate is significantly lower than those without HPS^30^. Therefore, we support the claim that additional scores should be given to PELD and MELD scores in HPS patients waiting for LT^30^. The PELD exception needs to be applied in Korea.

We found that PS/IVC was a risk factor for HPS in children with BA. Similar to our study, the presence of PS/IVC was more common in patients with HPS, and cirrhosis is not a requirement for the development of HPS in children^19,31^. However, the mechanism of the correlation between PS/IVC and HPS is still unknown^31^. Gupta et al. suggested that HPS is associated with a decrease in intrahepatic blood flow in patients with noncirrhotic portal hypertension. In patients with PS/IVC, the abnormality of intrahepatic blood flow was more severe, leading to a higher incidence of HPS^19^. The development of HPS in children with congenital portosystemic shunt and normal liver function suggests that HPS occurs as a consequence of bypassing normal intrahepatic blood flow and failure to metabolize vasodilatory mediators^32^.

Since there is no medical treatment to date for HPS, the only definite treatment is LT^22,33^. Our study shows that post-LT 5-year survival is excellent (100%), comparable to 88–93% reported in previous studies (2, 13). Among the 6 patients who underwent MAA and LT, 4 (67%) had severe HPS, and all 4 children survived. A Japanese study reported that the survival rate in the severe group was worse (40%) than those in the mild or moderate group^15^. In our study, the underlying disease in the severe group was BA. In the Japanese study, the major original disease was congenital portosystemic shunt in the severe group, suggesting that these patients might have pulmonary vessel malformation, which cannot be cured by LT^15^.

The limitations of this study are the small number of patients and the application of two methods to diagnose intrapulmonary vasodilation.

In conclusion, the prevalence of HPS in children with BA, portal vein agenesis, and portal vein thrombosis was 7%, 20%, and 4%, respectively. Only one of the total HPS patients died during the 10-year observation period, resulting in a 90% survival rate. Children with severe HPS showed excellent outcomes after LT. PS/IVC are a risk factor for the development of HPS in patients with BA. In addition, since the PELD and MELD scores alone cannot reflect the risk or aggravation of HPS, exceptions should be made for proper LT. Periodic pulse oximetry screening test for HPS in children with BA should be performed, especially in those with PS/IVC.
